# Global state of education-related inequality in COVID-19 vaccine coverage, structural barriers, vaccine hesitancy, and vaccine refusal: findings from the Global COVID-19 Trends and Impact Survey

**DOI:** 10.1016/S2214-109X(22)00520-4

**Published:** 2022-12-21

**Authors:** Nicole Bergen, Katherine Kirkby, Cecilia Vidal Fuertes, Anne Schlotheuber, Lisa Menning, Stephen Mac Feely, Katherine O'Brien, Ahmad Reza Hosseinpoor

**Affiliations:** a Department of Data and Analytics, WHO, Geneva, Switzerland; b Department of Immunisation, Vaccines and Biologicals, WHO, Geneva, Switzerland

## Abstract

**Background:**

COVID-19 vaccine coverage and experiences of structural and attitudinal barriers to vaccination vary across populations. Education-related inequality in COVID-19 vaccine coverage and barriers within and between countries can provide insight into the hypothesised role of education as a correlate of vaccine access and acceptability. We aimed to characterise patterns of within-country education-related inequality in COVID-19 vaccine indicators across 90 countries.

**Methods:**

This study used data from the University of Maryland Social Data Science Center Global COVID-19 Trends and Impact Survey. Data from 90 countries (more than 14 million participants aged 18 years and older) were included in our analyses. We assessed education-related inequalities globally, across country-income groupings, and nationally for four indicators (self-reported receipt of COVID-19 vaccine, structural barriers to vaccination, vaccine hesitancy, and vaccine refusal) for the study period June 1–Dec 31, 2021. We calculated an absolute summary measure of inequality to assess the latest situation of inequality and time trends and explored the association between government vaccine availability policies and education-related inequality.

**Findings:**

Nearly all countries had higher self-reported receipt of a COVID-19 vaccine among the most educated respondents than the least educated respondents. Education-related inequality in structural barriers, vaccine hesitancy, and vaccine refusal varied across countries, and was most pronounced in high-income countries, overall. Low-income and lower-middle-income countries reported widespread experiences of structural barriers and high levels of vaccine hesitancy alongside low levels of education-related inequality. Globally, vaccine hesitancy in unvaccinated people was higher among those with lower education and vaccine refusal was higher among those with higher education, especially in high-income countries. Over the study period, education-related inequalities in self-reported receipt of a COVID-19 vaccine declined, globally and across all country income groupings. Government policies expanding vaccine availability were associated with lower education-related inequality in self-reported receipt of vaccine.

**Interpretation:**

This study serves as a baseline for continued inequality monitoring and could help to inform targeted actions for the equitable uptake of vaccines.

**Funding:**

Gavi, the Vaccine Alliance.

## Introduction

The COVID-19 pandemic has exposed diverse forms of inequalities between and within countries, including inequalities pertaining to the receipt of, access to, and acceptance of COVID-19 vaccines. Inequalities in vaccine allocation across countries tend to favour high-income countries (HICs) and upper-middle-income countries (UMICs) over lower-income countries. As a result, the benefits of COVID-19 prevention and reduction in HICs and UMICs are not realised to the same extent in lower-income countries.^1^ *The WHO Strategy to Achieve Global COVID-19 Vaccination by Mid-2022* emphasised equity as a principle and called for monitoring of vaccine demand and uptake as a priority action.^2^

Vaccine uptake is affected by behavioural and social drivers.^3^ Barriers to vaccination have been broadly categorised as structural (systemic issues related to cost, convenience, and supply chain constraints) and attitudinal (relating to beliefs and perceptions that affect willingness to seek out or accept a vaccine).^4^ The relevance of various structural and attitudinal barriers is subject to fluctuation over time and across population subgroups as vaccination programmes and practices become embedded in societies. COVID-19 vaccine hesitancy has been shown to vary greatly across countries and among key groups, posing a threat to vaccination efforts.^5^

**Research in context**

**Evidence before this study**

We did an initial search of PubMed on Feb 25, 2022, for publications that quantified education-related inequalities in receipt of a COVID-19 vaccine, in structural barriers to COVID-19 vaccination, and in intention to receive a COVID-19 vaccine. Our search used MESH terms pertaining to the themes of health equity and COVID-19 vaccines, combined using the Boolean operators OR and AND (no date or language filters were applied). We reviewed titles and abstracts to identify relevant studies, and then conducted additional searches to identify other relevant papers, drawing from citation lists within these articles, suggested similar papers, and free text searches in Google Scholar and PubMed. Our searches predominantly yielded studies addressing intention to vaccinate, which were conducted with diverse populations and methods. There were few studies addressing education-related inequalities in receipt of vaccine or structural barriers to vaccination, as widespread vaccination programmes did not start until December, 2020, or later.

**Added value of this study**

This study used data from a large, multicountry online survey, encompassing 14 million respondents across 90 countries. To our knowledge, it is the first study of this scale dedicated to quantifying education-related inequality in COVID-19 vaccine indicators nationally, across country-income groupings, globally, and over time. Our analysis included four indicators pertaining to different aspects of vaccination (ie, self-report vaccine coverage as well as structural and attitudinal barriers), and assessed the state of inequality over a 7-month study period (June 1 to Dec 31, 2021). We also did a preliminary analysis of the association between government vaccine availability policies and education-related inequality in these indicators.

**Implications of all the available evidence**

In characterising education-related inequalities in four indicators at global, country-income group, and country levels, the results of this analysis are relevant to ongoing COVID-19 vaccination efforts. Our findings offer novel insight into the global state of education-related inequality in COVID-19 vaccine indicators, serving as a baseline for further study and helping to inform actions to ensure the benefits of vaccines can be equitably realised by all. Although cross-sectional analyses do not imply causation, the findings can provide insight into the hypothesised role of education as a determinant of these COVID-19 vaccine indicators. Together with other recent and emerging studies, this evidence will guide efforts to strengthen the equity-orientation of vaccination programmes and promote universal access to the benefits of vaccines.

Throughout 2021, vaccination programmes expanded as nearly every country introduced the COVID-19 vaccine. This constitutes a unique period for assessing and comparing global inequalities across settings and over time. An understanding of inequalities in vaccine uptake and barriers is an important starting point for identifying their root causes and developing remedial interventions.^6^ A focus on education-related inequality has several advantages over other dimensions of inequality such as economic status because information about education attainment is straightforward to gather from individuals, can be compared across settings, and has implications for the development of health programmes and policies.

We aimed to characterise patterns of within-country education-related inequality in COVID-19 vaccine indicators across 90 countries. Specifically, we report inequalities globally, across country-income groupings, and at the country level for indicators of self-reported receipt of a COVID-19 vaccine, structural barriers to vaccination, vaccine hesitancy, and vaccine refusal. We also explore time trends in inequality over a 7-month period in 2021, and the effect of country-level government policies on these indicators.

## Methods

### Data sources

Data were sourced from the largest global health survey to date, the University of Maryland Social Data Science Center Global COVID-19 Trends and Impact Survey (UMD Global CTIS).^7^, ^8^ UMD Global CTIS was a daily repeated cross-sectional survey conducted from April 23, 2020, to June 25, 2022, in more than 200 countries and territories (the number of countries and territories varied over time). We analysed data for the period June 1 to Dec 31, 2021.

Delivered through Facebook, the survey gathered data from more than 200 000 respondents aged 18 years and older each day. The survey was available in 56 languages, excluding only about 5% of the global Facebook userbase.^9^ The sampling design and weighting methodology of the UMD Global CTIS took into account sampling weights provided by Facebook to reduce non-response and coverage bias.^10^ Weighting for an individual was scaled to approximate the number of people in the adult population represented by that individual based on age, gender, location, and date (see the Statistical analysis section for more detail). Data about government vaccination policies were extracted from the Oxford COVID-19 Government Response Tracker (OxCGRT).^11^

Approval or exemption from the WHO Ethics Review Committee was not sought as the research was based on analysis of secondary data. The UMD Institutional Review Board (1587016–10) approved the UMD Global CTIS study. All respondents gave informed consent before participating in the survey. Informed consent was documented in the digital platform by the respondent (no witness required). None of the data that support the findings of this study included any identifiable human data.

### Study countries

Countries were considered for inclusion if they were WHO member states and if Facebook-provided sampling weights were available (n=109). The USA was not included in the UMD Global CTIS because data were collected through a separate survey.^12^ 19 countries were excluded from the analysis due to poor data representativeness (assessed as reporting fewer than 100 responses in at least half of the country age–sex subgroups by month). Additional sensitivity analysis was conducted to assess the effect of including and excluding these countries on the overall results. A link to the list of the 90 study countries and their corresponding sample sizes is provided in the appendix (p 2). Country income groupings are based on 2021 World Bank classifications.^13^ The low-income country (LIC) and lower-middle-income country (LMIC) groupings were combined due to the small number of LICs.

### Measures

This study included four binary outcome variables related to COVID-19 vaccination: self-reported receipt of COVID-19 vaccine; experienced structural barrier to vaccination; vaccine hesitancy; and vaccine refusal (table 1). Self-reported receipt of vaccine and structural barrier indicators were constructed on the basis of the total population of respondents, whereas the vaccine hesitancy and vaccine refusal indicators were constructed using the denominator of unvaccinated people only (that is, those who had not received a COVID-19 vaccine and did not have an appointment to receive the vaccine at the time of the survey). Missing answers were not included in the denominator.

**Table 1:** Survey questions related to COVID-19 vaccine outcome indicators

	**Question**	**Response options**	**Recording of response**
Self-reported receipt of COVID-19 vaccine*	Have you had a COVID-19 vaccination?	Yes or no	Yes or no
Experienced structural barrier to vaccination	Did you experience any of the following barriers to getting the COVID-19 vaccine?	I did not meet the eligibility requirements; there were no vaccines or appointments available; the available appointment times did not work for me; there were technical difficulties with the website or telephone line; I was unable to provide a required document; limited access to internet or telephone to schedule an appointment; difficulty travelling to a vaccination site; information not available in my native language; there is no-one to provide childcare while getting the vaccine; it was difficult to get time away from work or school; I could not get the type of vaccine I wanted; or none of the above	Recorded as structural barrier if response indicates any one or more barrier
Vaccine hesitancy (unvaccinated people)	If a vaccine to prevent COVID-19 were offered to you today, would you choose to get vaccinated?	Yes, definitely; yes, probably; no, probably not; or no, definitely not	Recorded as vaccine hesitancy for responses “yes, probably” or “no, probably not”
Vaccine refusal (unvaccinated people)	If a vaccine to prevent COVID-19 were offered to you today, would you choose to get vaccinated?	Yes, definitely; yes, probably; no, probably not; or no, definitely not	Recorded as vaccine refusal for response “no, definitely not”

These data do not represent official estimates of vaccine coverage for a given country or population subgroup.

* This indicator did not differentiate the number or type of COVID-19 vaccine doses.

The predictor variable was self-reported education level, measured as a categorical variable consisting of seven subgroups: no education, less than primary, primary, secondary, high school, college or university, and postgraduate. Control variables were: gender; age; place of residence (urban or rural); level of housing overcrowding (as a proxy for socioeconomic status^14^); the presence of at least one diagnosed health risk factor (asthma, lung disease, cancer, diabetes, high blood pressure, kidney disease, weak immune system, or obesity); and experience of COVID-like illness (fever plus either cough or difficulty breathing within the past 24 h).

For OxCGRT data, an ordinal scale was applied to reflect the country-level government policies for vaccine availability, specifying three population groups (key workers, clinically vulnerable people, or older people): 0 indicated no availability to any group; 1 indicated availability for one of the key groups; 2 indicated availability for two of the key groups; 3 indicated availability for all three key groups; 4 indicated availability for all three key groups plus partial additional availability; and 5 indicated universal availability. The mean policy score was calculated by country for each study month.

### Statistical analysis

Disaggregated data and summary measures of inequality were used to assess education-related inequality across the four outcome indicators for the overall study period and for each of the 7 months. Median values of country-level disaggregated estimates and inequality summary measures were calculated across all countries and by country income groups. Country-level disaggregated estimates were reported by education subgroups (no or primary education, secondary education, and higher education) if they included at least 100 responses for each subgroup for the study period.

A regression-based summary measure of inequality, slope index of inequality (SII), was calculated to evaluate absolute inequality (relative index of inequality, a relative summary measure of inequality, was also calculated, and is reported in the appendix pp 2, 4, 6–7). SII expresses the difference in the outcome variable between two extremes of the education distribution, accounting for the situation across the population.^15^, ^16^ This measure was applied for each study country and indicator; SII was calculated separately for males and females and for both groups combined.

SII was calculated at the individual level because this permitted a larger sample size and enabled controlling for other factors. SII of 0 indicates no inequality; SII above 0 denotes higher indicator prevalence among people with more education, and SII below 0 denotes higher indicator prevalence among people with less education.

Adjusted SII was calculated for the overall period (small sample sizes prevented models from converging for monthly data in many countries). In the first model (adjusted) we controlled for individual sociodemographic characteristics (age, gender, place of residence, and household overcrowding). The second model additionally controlled for the presence of health risk factors and COVID-19-like symptoms (adjusted2). A Poisson regression model with a robust variance was used to generate the SII values and corresponding 95% CIs. We used Poisson regression with the robust variance option to give an unbiased standard error; this provides more accurate estimates compared with a logit model when the outcome has a high prevalence.^17^

Additional analysis investigated the association between education-related inequality and country-level government policies. Government policy scores were correlated—globally and by income group—against the national prevalence and education-related inequality (crude SII) for the selected indicator using Spearman correlations. Results were not reported when country-level SII values included both negative and positive values.

All analyses were weighted to account for the survey sampling design and non-response bias. The weights applied in our analysis were derived from those created and provided by the UMD Global CTIS group at the time of analysis. Briefly, this approach used inverse propensity score weighting to adjust for non-response error to make the sample more representative of the sampling frame of Facebook app users (using covariates available through internal Facebook data, such as self-reported age, gender, subnational region, and other attributes found in the past to correlate with survey outcomes).^10^, ^18^ Then, to improve representation of the entire population in a country, a second step of poststratification using publicly available population benchmarks and the inverse propensity score weighting output weights were applied as input weights. Following recommendations by Barkay and colleagues,^10^ we used survey response data to check representation of key demographic variables against population benchmarks and applied additional poststratification steps to address differences. In addition to using the weights provided within the UMD Global CTIS, we applied poststratification weights to adjust for differences in age–sex population distribution patterns compared with population benchmarks, drawing from population distributions in the UN Population Division 2019 World Population Prospects.^19^ This approach was discussed with the UMD Global CTIS analysis group to verify suitability for our purposes (and the UMD Global CTIS analysis group has since integrated similar poststratification weights into their weighting methodology^18^). 95% CIs were used to ascertain statistical significance. All analysis were conducted using STATA version 16.1.

### Role of the funding source

The funder of the study had no role in study design, data collection, data analysis, data interpretation, or writing of the report.

## Results

This study included approximately 14 million adult participants across 90 countries (33 HICs, 29 UMICs, 24 LMICs, and four LICs). Globally across all study countries, the median national self-reported receipt of a COVID-19 vaccine for the study period was 75·2% (95% CI 72·1–79·8) and the median proportion of people who experienced a structural barrier to vaccination was 23·9% (20·4–27·4; table 2). The median national self-reported vaccination prevalence was significantly higher in HICs than in UMICs or LICs and LMICs, and the median national prevalence of experiencing a structural barrier was significantly lower in HICs than in UMICs or LICs and LMICs. Globally, the median prevalence of vaccine hesitancy among unvaccinated people was 39·9% (95% CI 37·0–41·7) and median vaccine refusal among unvaccinated people was 28·8% (24·5–37·2). Compared with UMICs and LICs and LMICs, HICs reported lower vaccine hesitancy, and higher vaccine refusal overall.

**Table 2:** COVID-19 vaccine indicators by education level: median national prevalence, disaggregated estimates, and crude and adjusted SII, globally and by country income group

	**Number of countries**	**Median national prevalence (95% CI)**	**Median disaggregated data (95% CI)**			**Median SII, percentage points (95% CI)**		
			No or primary education	Secondary education	Higher education	Unadjusted	Adjusted	Adjusted2
**Self-reported receipt of COVID-19 vaccine**								
Global	90	75·2% (72·1–79·8)	67·7% (60·0–73·0)	71·4% (67·4–76·1)	79·1% (75·6–82·4)	16·4 (13·4 to 19·3)	11·3 (9·1 to 12·7)	11·9 (10·2 to 13·4)
High-income countries	33	86·0% (81·6–87·5)	81·0% (78·3–87·1)	83·8% (78·8–86·0)	87·4% (83·4–89·0)	10·3 (7·3 to 12·2)	6·9 (5·6 to 8·7)	6·9 (6·0 to 8·3)
Upper-middle-income countries	29	71·5% (65·6–77·9)	57·6% (52·5–72·3)	66·7% (58·8–73·9)	75·5% (68·1–82·1)	21·4 (17·3 to 24·5)	14·3 (11·9 to 16·7)	14·5 (12·9 to 17·1)
Low-income and lower-middle-income countries	28	58·1% (51·0–73·0)	50·7% (41·8–59·6)	51·2% (41·9–67·0)	63·9% (53·0–75·6)	19·5 (14·2 to 26·6)	13·9 (9·3 to 16·8)	13·3 (11·1 to 18·1)
**Experienced structural barrier to vaccination**								
Global	90	23·9% (20·4–27·4)	25·3% (21·8–28·5)	23·6% (19·6–26·6)	23·1% (20·2–25·7)	−3·7 (−6·4 to −1·5)	3·8 (2·6 to 4·7)	3·6 (2·4 to 5·4)
High-income countries	33	15·0% (14·1–17·1)	12·6% (11·6–14·0)	13·5% (12·5–15·1)	16·9% (15·4–19·8)	2·2 (0·2 to 4·8)	4·8 (3·6 to 8·6)	5·3 (3·4 to 9·0)
Upper-middle-income countries	29	24·0% (21·5–29·9)	27·0% (22·5–31·2)	23·6% (21·3–30·0)	23·4% (21·5–27·3)	−5·5 (−8·5 to −3·5)	3·7 (2·0 to 7·5)	3·1 (1·2 to 6·3)
Low-income and lower-middle-income countries	28	34·6% (29·3–38·9)	38·3% (32·9–45·5)	33·5% (28·3–40·5)	34·1% (28·3–38·1)	−8·3 (−11·0 to −6·5)	0·5 (−1·3 to 4·6)	1·1 (−1·4 to 6·6)
**Vaccine hesitancy (unvaccinated people)**								
Global	90	39·9% (37·0–41·7)	38·7% (37·3–40·1)	41·1% (39·0–43·0)	38·2% (35·7–41·7)	−4·1 (−6·0 to −1·0)	−4·6 (−7·0 to −3·3)	−3·8 (−6·4 to −1·4)
High-income countries	33	34·9% (33·7–37·7)	38·8% (35·5–41·4)	39·1% (34·8–42·4)	33·1% (31·4–36·2)	−7·0 (−8·2 to −2·8)	−6·7 (−9·5 to −4·0)	−6·4 (−10·5 to −2·3)
Upper-middle-income countries	29	41·7% (37·7–44·8)	39·2% (37·1–41·2)	43·5% (40·3–45·2)	40·7% (34·7–44·6)	−5·7 (−9·6 to 0·6)	−4·9 (−9·6 to −1·8)	−4·9 (−8·6 to −0·2)
Low-income and lower-middle-income countries	28	41·4% (39·2–46·5)	37·8% (32·5–41·6)	40·9% (38·3–44·0)	44·0% (40·2–46·8)	1·2 (−2·8 to 7·0)	2·1 (−4·4 to 5·3)	1·8 (−4·5 to 7·5)
**Vaccine refusal (unvaccinated people)**								
Global	90	28·8% (24·5–37·2)	27·4% (23·8–38·0)	26·7% (20·0–36·3)	28·3% (24·3–37·6)	6·0 (4·4 to 8·0)	4·0 (2·5 to 6·3)	2·5 (1·4 to 4·8)
High-income countries	33	47·4% (39·0–59·1)	42·8% (31·2–49·0)	46·9% (37·0–57·4)	50·7% (40·5–60·7)	8·5 (6·0 to 12·4)	9·6 (5·7 to 10·7)	8·1 (3·1 to 10·1)
Upper-middle-income countries	29	27·3% (22·5–33·6)	24·9% (17·4–35·0)	24·3% (14·1–34·7)	27·7% (21·5–34·2)	6·0 (3·2 to 10·7)	2·9 (−0·0 to 7·3)	1·5 (−0·1 to 5·7)
Low-income and lower-middle-income countries	28	18·5% (15·1–24·2)	19·5% (16·1–24·7)	17·3% (12·9–21·9)	17·5% (14·8–24·2)	2·2 (−0·9 to 5·0)	1·0 (−1·6 to 3·1)	−0·3 (−2·0 to 1·9)

Medians are based on countries with sample sizes of at least 100 in each education subgroup. Disaggregated data estimates excluded countries with insufficient sample size; estimates based on 87 countries for experienced structural barrier (28 upper-middle-income countries and 26 low-income and lower-middle-income countries) and 86 countries for vaccine hesitancy and vaccine refusal (31 high-income and 26 low-income and lower-middle-income countries). SII of 0 indicates no inequality; SII above 0 denotes higher indicator prevalence among people with more education, and SII below 0 denotes higher indicator prevalence among people with less education. The first adjusted model (adjusted) controlled for individual sociodemographic characteristics (age, gender, place of residence, and household overcrowding). The second adjusted model (adjusted2) controlled for these characteristics plus the presence of health risk factors and COVID-19-like symptoms. SII=slope index of inequality.

In general, patterns of education-related inequality were similar for males and females (appendix p 6); therefore, we report results from combined data for males and females.

**Figure 1:**
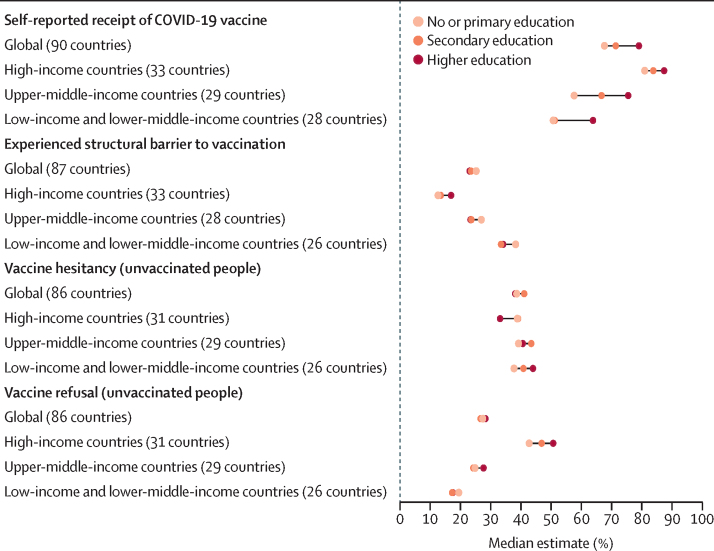
COVID-19 vaccine indicators by education level, globally and by country income group Data are median disaggregated estimates. Medians are based on countries with sample sizes of at least 100 in each education subgroup.

Globally, there was a stepwise pattern of inequality across the three education subgroups for median self-reported receipt of COVID-19 vaccine (favouring those with higher education), which was also evident across HICs and, to a greater extent, in UMICs (figure 1). In LICs and LMICs, the median self-reported receipt of a COVID-19 vaccine was higher among the most educated subgroup, and equally low in the two less educated subgroups. There were no clear patterns of education-related inequality at the global level for the structural barrier, vaccine hesitancy, and vaccine refusal indicators. In HICs, the median experience of a structural barrier was significantly higher among the higher education subgroup than the other two education subgroups (table 2). There were some variations observed across education subgroups within country income groups for vaccine hesitancy and vaccine refusal. Country-level information, including sample sizes and national and disaggregated estimates for each indicator, is available via the link in the appendix (p 2).

**Figure 2:**
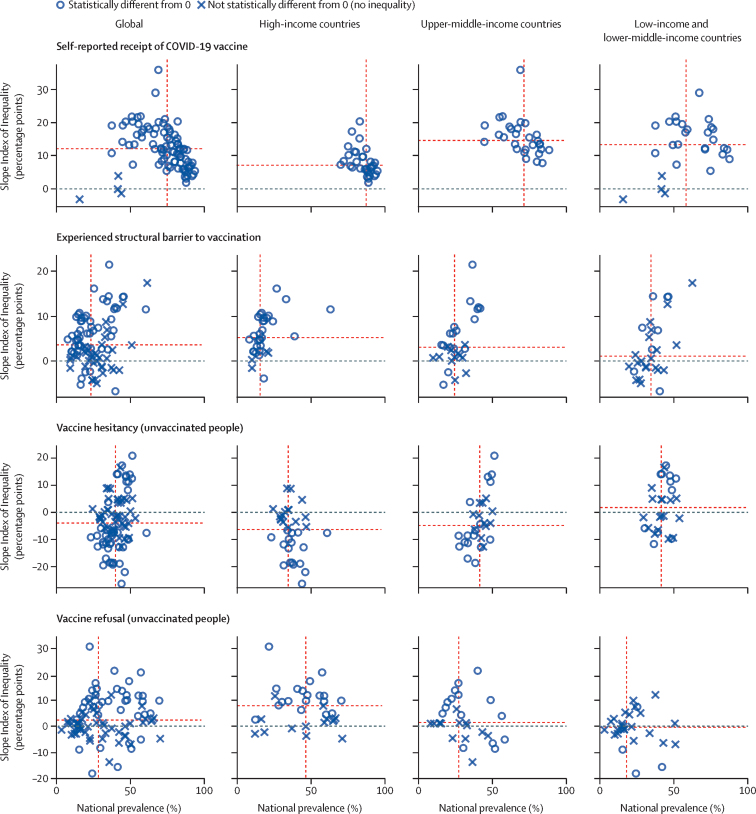
Education-related inequality in COVID-19 vaccine indicators, globally and by country income group Data are adjusted SIIs compared with national prevalence. Blue symbols represent countries. Red dashed lines show the medians across countries. Grey dashed line shows no inequality (zero). Positive SII values denote higher indicator prevalence among people with more education, and negative SII values denote higher indicator prevalence among people with less education. SII is adjusted for individual sociodemographic characteristics (age, gender, place of residence, and household overcrowding), and the presence of health risk factors and COVID-like symptoms. SII=slope index of inequality.

Summary measures of inequality, accounting for the situation across the entire population, further quantified these patterns (figure 2, with country-level findings available via the link in the appendix p 2, relative index of inequality shown in the appendix pp 4, 7, and crude SII shown in the appendix p 3).

Education-related inequality in self-reported receipt of a COVID-19 vaccine favoured the most educated individuals, globally (median adjusted SII 11·9 percentage points, 95% CI 10·2–13·4) and in each country income group (table 2). The median level of inequality was more pronounced in UMICs and LICs and LMICs than in HICs (where the median national prevalence was also higher). Adjusting for other factors, nearly all countries showed significant education-related inequality favouring the most educated respondents (except Ethiopia, Kyrgyzstan, Nigeria, and Yemen, where SII was non-significant and national prevalence estimates were less than 45%; appendix p 2). More than a third of countries (31 [34%] of 90) reported absolute education-related inequality in self-reported receipt of vaccine of 15 percentage points or more, adjusting for other factors (appendix p 2).

Globally, the median level of education-related inequality in structural barriers indicated more barrier experiences among those with less education than those with more education (crude SII –3·7 percentage points; 95% CI –6·4 to –1·5), although the direction of inequality reversed when adjusting for other sociodemographic, health risk factors and COVID-19 symptoms (adjusted SII 3·6 percentage points; 95% CI 2·4 to 5·4; table 2). Median inequalities were low, overall, in HICs and UMICs (crude and adjusted median SIIs of around 5 percentage points or less). In LICs and LMICs, median education-related inequality was non-significant when adjusting for other factors (table 2).

Education-related inequality in vaccine hesitancy among unvaccinated people was present globally and among HICs and UMICs, with a higher level of hesitancy among the least educated respondents, although by fairly small margins (table 2). Overall education-related inequality in vaccine hesitancy in LICs and LMICs was small and not statistically significant (table 2).

Around half of HICs (17 [52%] of 33) reported significantly higher levels of hesitancy among the least educated respondents: adjusted SII values ranged from around –6 to –7 percentage points in Canada, Italy, Japan, and Spain to near –20 percentage points in Ireland, Sweden, and Uruguay, and less than –20 percentage points in Finland and Singapore. The remaining HICs reported no significant inequality in vaccine hesitancy (appendix p 2).

UMICs and LICs and LMICs showed more varied directionality of inequality than HICs, with some countries reporting higher hesitancy among the most educated respondents, and some reporting higher hesitancy among the least educated respondents (figure 2). For instance, in Albania, Ghana, and Pakistan, vaccine hesitancy was at least 15 percentage points higher among the most educated respondents than the least educated respondents. Guatemala and Paraguay also reported a gap of more than 15 percentage points, with higher hesitancy among the least educated respondents than the most educated respondents (appendix p 2).

In contrast to vaccine hesitancy, vaccine refusal tended to be more common among the most educated respondents than the least educated respondents, globally and especially in HICs (table 2). There was no overall education-related inequality across UMICs and LICs and LMICs, adjusting for other factors. Although all HICs reported either no significant inequality or higher vaccine refusal among the most educated respondents, country patterns of inequality in UMICs and LICs and LMICs were varied (appendix p 2).

**Figure 3:**
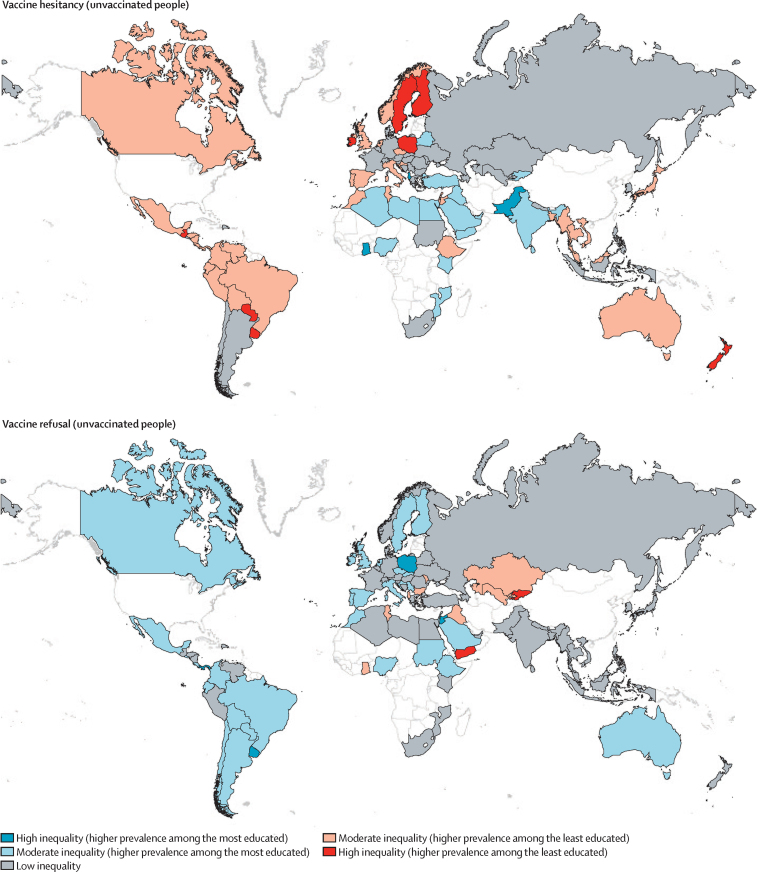
Education-related inequality in COVID-19 vaccine indicators in 90 study countries Data are adjusted SIIs. High inequality denotes an absolute SII of 15 percentage points or more; moderate inequality denotes an absolute SII of 5–15 percentage points; low inequality denotes an absolute SII of less than 5 percentage points. SII is adjusted for individual sociodemographic characteristics (age, gender, place of residence, and household overcrowding), and the presence of health risk factors and COVID-like symptoms. SII=slope index of inequality.

Countries in the American and European continents tended to report higher vaccine hesitancy among the least educated respondents (or low inequality), whereas countries in the African and Asian continents tended to report higher vaccine hesitancy among the most educated respondents (or low inequality), with some exceptions (figure 3). Among many countries in the American and European continents, the opposite direction of inequality was observed for vaccine refusal, with higher refusal among the most educated respondents. In many African and Asian countries, there were no education-related inequalities in vaccine refusal, with other countries showing different directions of inequality. Maps of country-level education-related inequality in experiences of structural barriers to vaccination are shown in the appendix (p 5).

**Figure 4:**
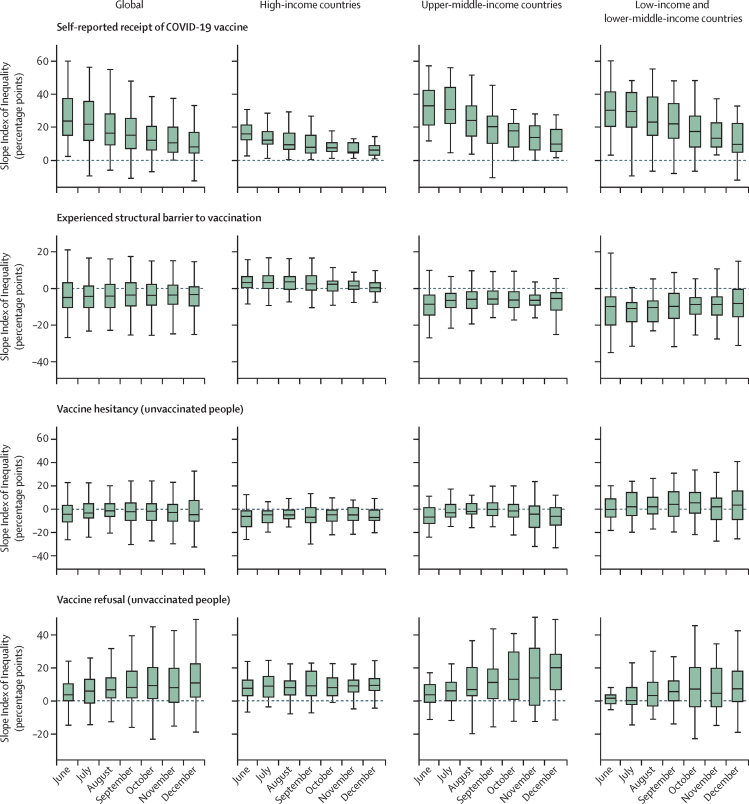
Change over time in education-related inequality in COVID-19 vaccine indicators, globally and by country income group Data are crude (unadjusted) SIIs. The centre line of the box plot indicates the median, the shaded box indicates the IQR (middle 50% of study country estimates), and the top and bottom lines indicate the minimum and maximum values. Grey dashed line shows no inequality (zero). Positive SII values denote higher indicator prevalence among people with more education, and negative SII values denote higher indicator prevalence among people with less education. SII=slope index of inequality.

Median education-related inequalities in self-reported receipt of COVID-19 vaccine declined globally and across all country income groups during the 7-month study period (figure 4). Inequalities in structural barriers to vaccination declined, globally and in UMICs and LICs and LMICs, particularly from June to September. Vaccine hesitancy did not show obvious patterns of change in the median level of education-related inequality over time. For vaccine refusal, median education-related inequality showed small increases over time globally, and especially in UMICs, with increasing variation in country inequality estimates in UMICs and LICs and LMICs. In HICs, vaccine refusal was consistently higher among the most educated respondents than the least educated respondents across all 7 months (figure 4).

Greater vaccine availability, as per government policies, was associated with higher national levels of self-reported receipt of COVID-19 vaccine and lower education-related inequality (crude SII), globally and in all country income groups (appendix p 8). As government policies specified increased vaccine availability, the national prevalence of experiencing a structural barrier to vaccination declined; however, data from HICs and UMICs suggest that government policies were not associated with education-related inequalities in this indicator (appendix p 8). Similarly, government policies were not associated with education-related inequalities in vaccine hesitancy and refusal in HICs and UMICs (appendix p 8).

## Discussion

To our knowledge, this was the largest multicountry study of education-related inequalities in COVID-19 vaccine indicators. Whereas nearly all countries reported significant inequality in self-reported receipt of vaccine favouring the most educated respondents, the direction of inequalities in the three structural and attitudinal barrier indicators varied across countries. Overall, HICs showed pronounced inequalities for these three indicators, and inequalities across LICs and LMICs were low overall. Given that LICs and LMICs had widespread structural barriers and high levels of vaccine hesitancy but generally low levels of education-related inequality, these barriers were commonly reported, regardless of education level.

It is difficult to directly compare our results with other sources due to the paucity of multicountry studies on this topic, but also due to the time-bound nature of the analysis. Self-reported receipt of a COVID-19 vaccine is a cumulative measure that increases over time. Structural barriers and attitudes about vaccines also fluctuate over time.^20^ Nevertheless, there are previous and ongoing efforts to quantify and compare similar COVID-19 indicators across countries that help to situate the findings of this analysis. The WHO country-income-level estimates of full COVID-19 vaccination status also suggest higher vaccination rates among higher-income country groupings,^21^ which is in keeping with our finding of overall lower structural barriers in HICs and UMICs. Several multicountry studies assessing COVID-19 vaccine acceptance before or in the very early stages of vaccine roll-out reported divergent directionality of education-related inequality across countries.^22^, ^23^, ^24^

Our findings suggest that in many countries, but especially HICs, structural barriers to vaccination were often reported by those with higher levels of education, despite self-reported receipt of vaccine being higher among more educated respondents. This result might be partially explained by the inclusion of the survey response option “I could not get the type of vaccine I wanted” as part of the indicator (an additional analysis excluding this response showed overall lower levels of education-related inequality in HICs). Respondents in HICs with higher levels of education might have had more information and options available about vaccine type compared with respondents with lower levels of education. Choices about vaccine type might be less applicable in other contexts.

We reported vaccine hesitancy and refusal indicators among unvaccinated people, which allowed for a more focused depiction of education-related inequality among those likely to be targeted by public health communication and vaccination campaigns. We identified variable patterns across countries, although our findings additionally point to overarching trends across country income groups. Notably, among unvaccinated people in HICs, vaccine hesitancy was skewed towards lower levels of education, whereas those who refused vaccines were more likely to have higher levels of education. This pattern was also evident, to a lesser extent, in UMICs and not in LICs and LMICs (which might be explained by the lower levels of self-reported receipt of vaccine and resulting larger denominator populations in LICs and LMICs). These findings are relevant for actions to manage and dismantle the COVID-19 misinformation and disinformation that have undermined responses to the COVID-19 pandemic. Previous research has shown that exposure to misinformation about COVID-19 has differential effects on vaccination intent based on education level and other sociodemographic characteristics.^25^

Vaccine hesitancy, a phenomenon of growing interest before and throughout the COVID-19 pandemic, is inherently complex and context-specific, and subject to variation across time, place, and vaccines.^26^ Our indicator of vaccine hesitancy aligns with existing conceptual frameworks that situate hesitancy as a continuum between vaccine refusal and acceptance, recognising the distinction from vaccine refusal.^27^ We reported different directionality of education-related inequality in vaccine hesitancy versus refusal, and different trends over time. Previous systematic reviews have found that survey wording and answer options influence responses to questions about vaccine intention,^20^, ^28^ and thus additional evidence is required to corroborate our findings. Recent tools developed by WHO provide guidance on the collection and use of data about behavioural and social drivers of vaccine uptake,^3^ with the Strategic Advisory Group of Experts on Immunization recommending that all countries take steps to expand this knowledge base to strengthen programme planning and implementation.^29^

Whereas previous studies have examined the mediating role of vaccination policy in explaining different COVID-19 vaccination coverage across country-income groupings,^30^ our findings regarding the association between government policies and education-related inequality constitute a novel addition to the literature. Our preliminary findings showed that, as government policies expand vaccine availability, education-related inequalities in self-reported receipt of vaccine decline, globally and across all country income groups. This finding might be explained, in part, by health-care workers, who have higher levels of education than the general population, being among the first to be eligible for vaccination. However, education-related inequalities in structural barriers, vaccine hesitancy, and vaccine refusal in HICs and UMICs were not affected. These results suggest a need for further analyses, including at the country level, to explore these patterns and their drivers.

Our analysis is subject to some limitations, including those common to digital surveys and UMD Global CTIS.^31^ Our data, collected using surveys delivered through Facebook, are not necessarily representative of larger populations and might contain non-response and sampling biases. Data quality might be affected by social desirability bias or intentional misreporting (ie, deliberate trolling, which might target large, online surveys on controversial topics) and biases might be compounded by large sample sizes. Cross-sectional analyses do not imply causation. The estimates reported here are not official WHO estimates and are not directly comparable with official estimates. We did not report separate estimates for the LIC group due to the low response rate in these countries. Other data suggest markedly lower COVID-19 vaccination in this group than the LMIC group,^21^ thus our combined estimates across LICs and LMICs might conceal differences between the two groups.

The COVID-19 pandemic is unprecedented in its global scope and sociopolitical context, among other factors. Dedicated research examining inequalities in vaccine-related experiences and intentions is warranted to ensure that the benefits of vaccines are equitably available to all. This study, the first to characterise education-related inequality in COVID-19 vaccine indicators on a global scale, provides a basis for subsequent investigations into sources of inequities, their interactions, and the pluralistic approaches to address them across diverse population groups.

## Data sharing

This paper is based on secondary analysis of the University of Maryland (UMD) Global COVID-19 Trends and Impact Survey dataset, which is available on request from UMD (https://covidmap.umd.edu/fbsurvey/). Survey microdata are not publicly available because survey participants only consented to public disclosure of aggregate data, and because the legal agreement with Facebook governing operation of the survey prohibits disclosure of microdata without confidentiality protections for respondents. Deidentified microdata are available to researchers under a data use agreement that protects the confidentiality of respondents. Country-level aggregate weekly and monthly estimates for select indicators are also published by the UMD (https://covidmap.umd.edu/api.html). The aggregate estimates and code used in this analysis are available from the corresponding author upon reasonable request. The OxCGRT is publicly available online (https://www.nature.com/articles/s41562-021-01079-8).

## Declaration of interests

We declare no competing interests. The views expressed in this Article are those of the authors and do not necessarily represent the views or policies of WHO.

## References

[1] Ye Y, Zhang Q, Wei X, Cao Z, Yuan H-Y, Zeng DD (2022). Equitable access to COVID-19 vaccines makes a life-saving difference to all countries. Nat Hum Behav.

[2] WHO (2021).

[3] WHO (2022).

[4] Zhang Y, Fisk RJ (2021). Barriers to vaccination for coronavirus disease 2019 (COVID-19) control: experience from the United States. Glob Health J.

[5] Sallam M (2021). COVID-19 vaccine hesitancy worldwide: a concise systematic review of vaccine acceptance rates. Vaccines (Basel).

[6] Ismail SJ, Tunis MC, Zhao L, Quach C (2021). Navigating inequities: a roadmap out of the pandemic. BMJ Glob Health.

[7] University of Maryland (2022). The University of Maryland Social Data Science Center Global COVID-19 Trends and Impact Survey in partnership with Facebook. https://covidmap.umd.edu/.

[8] Kreuter F, Barkay N, Bilinski A (2020). Partnering with a global platform to inform research and public policy making. Surv Res Methods.

[9] Astley CM, Tuli G, Mc Cord KA (2021). Global monitoring of the impact of the COVID-19 pandemic through online surveys sampled from the Facebook user base. Proc Natl Acad Sci USA.

[10] Barkay N, Cobb C, Eilat R (2020). Weights and methodology brief for the COVID-19 Symptom Survey by University of Maryland and Carnegie Mellon University, in partnership with Facebook. arXiv.

[11] Blavatnik School of Government, University of Oxford (2022). COVID-19 government response tracker. https://www.bsg.ox.ac.uk/research/research-projects/covid-19-government-response-tracker.

[12] Carnegie Mellon University Delphi Group Delphi's COVID-19 Trends and Impact Surveys (CTIS). https://delphi.cmu.edu/covid19/ctis/.

[13] The World Bank (2021). World Bank country and lending groups. https://datahelpdesk.worldbank.org/knowledgebase/articles/906519-world-bank-country-and-lending-groups.

[14] Cable N, Sacker A (2019). Validating overcrowding measures using the UK Household Longitudinal Study. SSM Popul Health.

[15] Schlotheuber A, Hosseinpoor AR (2022). Summary measures of health inequality: a review of existing measures and their application. Int J Environ Res Public Health.

[16] WHO (2013).

[17] Barros AJD, Hirakata VN (2003). Alternatives for logistic regression in cross-sectional studies: an empirical comparison of models that directly estimate the prevalence ratio. BMC Med Res Methodol.

[18] COVID-19 Trends and Impact Survey (2022). Methodology report for the COVID-19 trends and impact survey: version 1. The Delphi Group at Carnegie Mellon University and University of Maryland Social Data Science Center COVID-19 Trends and Impact Survey (CTIS), in partnership with Meta. https://dataforgood.facebook.com/dfg/resources/CTIS-methodology-report.

[19] UN Department of Economic and Social Affairs World population prospects. https://population.un.org/wpp/.

[20] Robinson E, Jones A, Lesser I, Daly M (2021). International estimates of intended uptake and refusal of COVID-19 vaccines: a rapid systematic review and meta-analysis of large nationally representative samples. Vaccine.

[21] WHO (2022). WHO coronavirus (COVID-19) dashboard. https://covid19.who.int/.

[22] Solís Arce JS, Warren SS, Meriggi NF (2021). COVID-19 vaccine acceptance and hesitancy in low- and middle-income countries. Nat Med.

[23] Lazarus JV, Wyka K, Rauh L (2020). Hesitant or not? The association of age, gender, and education with potential acceptance of a COVID-19 vaccine: a country-level analysis. J Health Commun.

[24] Aw J, Seng JJB, Seah SSY, Low LL (2021). COVID-19 vaccine hesitancy—a scoping review of literature in high-income countries. Vaccines (Basel).

[25] Loomba S, de Figueiredo A, Piatek SJ, de Graaf K, Larson HJ (2021). Measuring the impact of COVID-19 vaccine misinformation on vaccination intent in the UK and USA. Nat Hum Behav.

[26] MacDonald NE (2015). Vaccine hesitancy: definition, scope and determinants. Vaccine.

[27] Dubé E, Laberge C, Guay M, Bramadat P, Roy R, Bettinger J (2013). Vaccine hesitancy: an overview. Hum Vaccin Immunother.

[28] Lin C, Tu P, Beitsch LM (2020). Confidence and receptivity for COVID-19 vaccines: a rapid systematic review. Vaccines (Basel).

[29] WHO Meeting of Strategic Advisory Group of Experts on Immunization, October 2021: conclusions and recommendations. https://www.who.int/publications/i/item/who-wer9650-613-632.

[30] Duan Y, Shi J, Wang Z, Zhou S, Jin Y, Zheng Z-J (2021). Disparities in COVID-19 vaccination among low-, middle-, and high-income countries: the mediating role of vaccination policy. Vaccines (Basel).

[31] University of Maryland Center for Geospatial Information Science (2021). Survey limitations. https://gisumd.github.io/COVID-19-API-Documentation/docs/survey_limitations.html.

