# Supplementary material for: Global state of education-related inequality in COVID-19 vaccine coverage, structural barriers, vaccine hesitancy, and vaccine refusal: findings from the Global COVID-19 Trends and Impact Survey
Source: Lancet Glob Health. 2022 Dec 21;11(2):e207–17. doi: 10.1016/S2214-109X(22)00520-4 (PMC9771421; doi:10.1016/S2214-109X(22)00520-4)
Supplement: Supplementary appendix [file mmc1.pdf]

THE LANCET
Global Health

Supplementary appendix

This appendix formed part of the original submission and has been peer reviewed. We post it as supplied by the authors.

Supplement to: Bergen N, Kirkby K, Fuertes CV, et al. Global state of education-related inequality in COVID-19 vaccine coverage, structural barriers, vaccine hesitancy, and vaccine refusal: findings from the Global COVID-19 Trends and Impact Survey. *Lancet Glob Health* 2022; published online Dec 21. https://doi.org/10.1016/S2214-109X(22)00520-4.

Supplementary materials

Contents

Interactive table 1. COVID-19 vaccine indicators by education level: national prevalence, disaggregated estimates, crude and adjusted slope index of inequality (SII) and relative index of inequality (RII), across 90 study countries 2

Figure S1. Education-related inequality in COVID-19 vaccine indicators: unadjusted slope index of inequality compared to national prevalence, globally and by country income group (UMD Global CTIS, June–December 2021) 3

Figure S2. Education-related inequality in COVID-19 vaccine indicators: adjusted relative index of inequality compared to national prevalence, globally and by country income group (UMD Global CTIS, June–December 2021) 4

Figure S3. Education-related inequality in experience of structural barriers to vaccination: unadjusted and adjusted slope index of inequality in 90 study countries (UMD Global CTIS, June–December 2021)5

Table S1. Education-related inequality in COVID-19 vaccine indicators: adjusted slope index of inequality (percentage points) and relative index of inequality, globally and by country income group for males and females (UMD Global CTIS, June–December 2021) 6

Table S2. COVID-19 vaccine indicators by education level: median relative index of inequality (RII), globally and by country income group (UMD Global CTIS, June–December 2021) 7

Table S3. Associations between government vaccine availability policies and self-reported receipt of COVID-19 (national prevalence and education-related inequality) (UMD Global CTIS, June–December 2021) 8

Interactive table 1. COVID-19 vaccine indicators by education level: national prevalence, disaggregated estimates, crude and adjusted slope index of inequality (SII) and relative index of inequality (RII), across 90 study countries

Available online from: COVID-19 vaccination indicators by education level: Interactive table | Tableau Public

Figure S1. Education-related inequality in COVID-19 vaccine indicators: unadjusted slope index of inequality compared to national prevalence, globally and by country income group (UMD Global CTIS, June–December 2021)

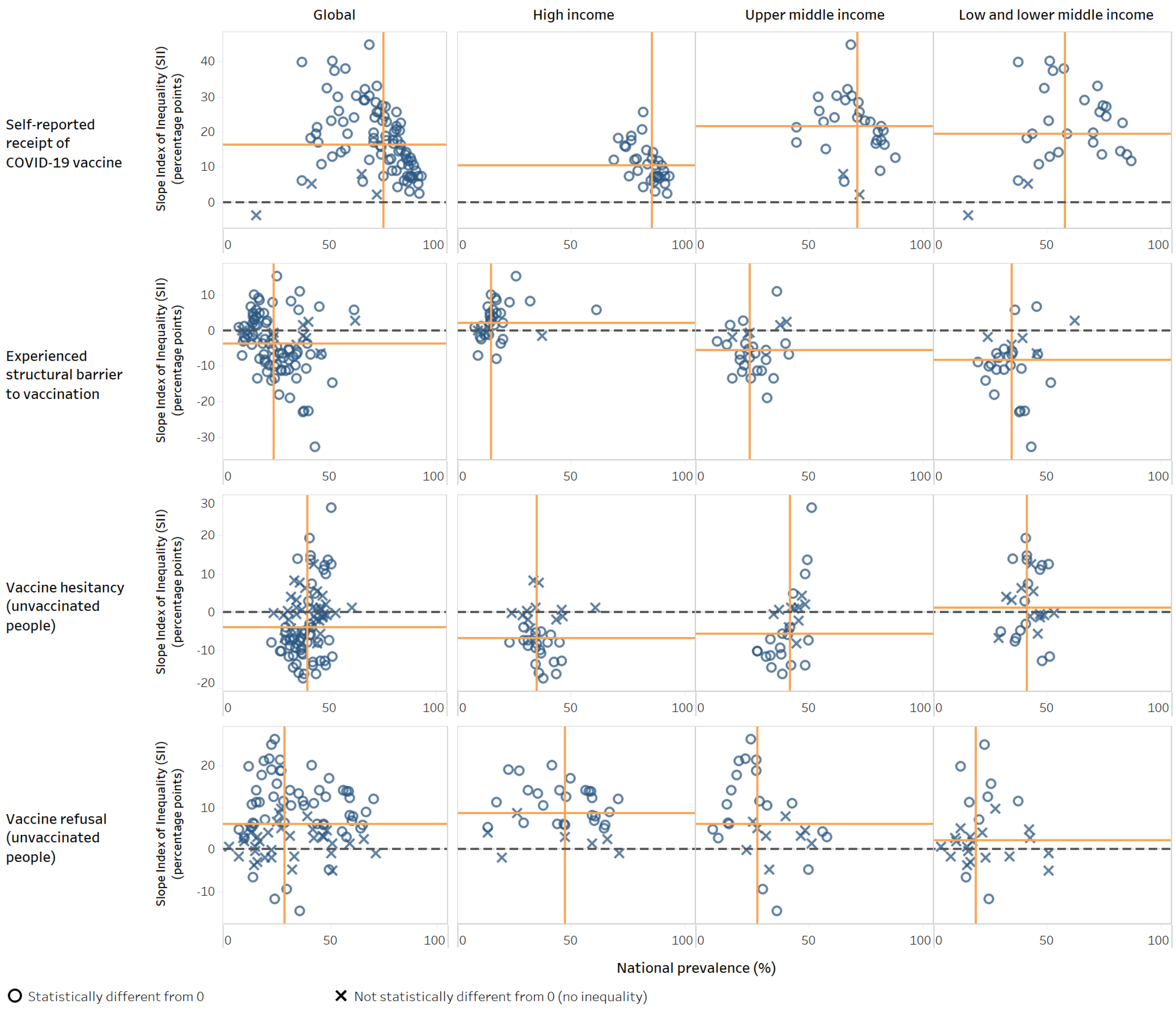

SII: Slope index of inequality. UMD Global CTIS: University of Maryland Social Data Science Center Global COVID-19 Trends and Impact Survey.
Notes: Blue symbols represent countries. Orange lines show the medians across countries. Black dashed line shows no inequality (zero). Positive SII values denote higher indicator prevalence among people with more education, and negative SII values denote higher indicator prevalence among people with less education.

Figure S2. Education-related inequality in COVID-19 vaccine indicators: adjusted relative index of inequality compared to national prevalence, globally and by country income group (UMD Global CTIS, June–December 2021)

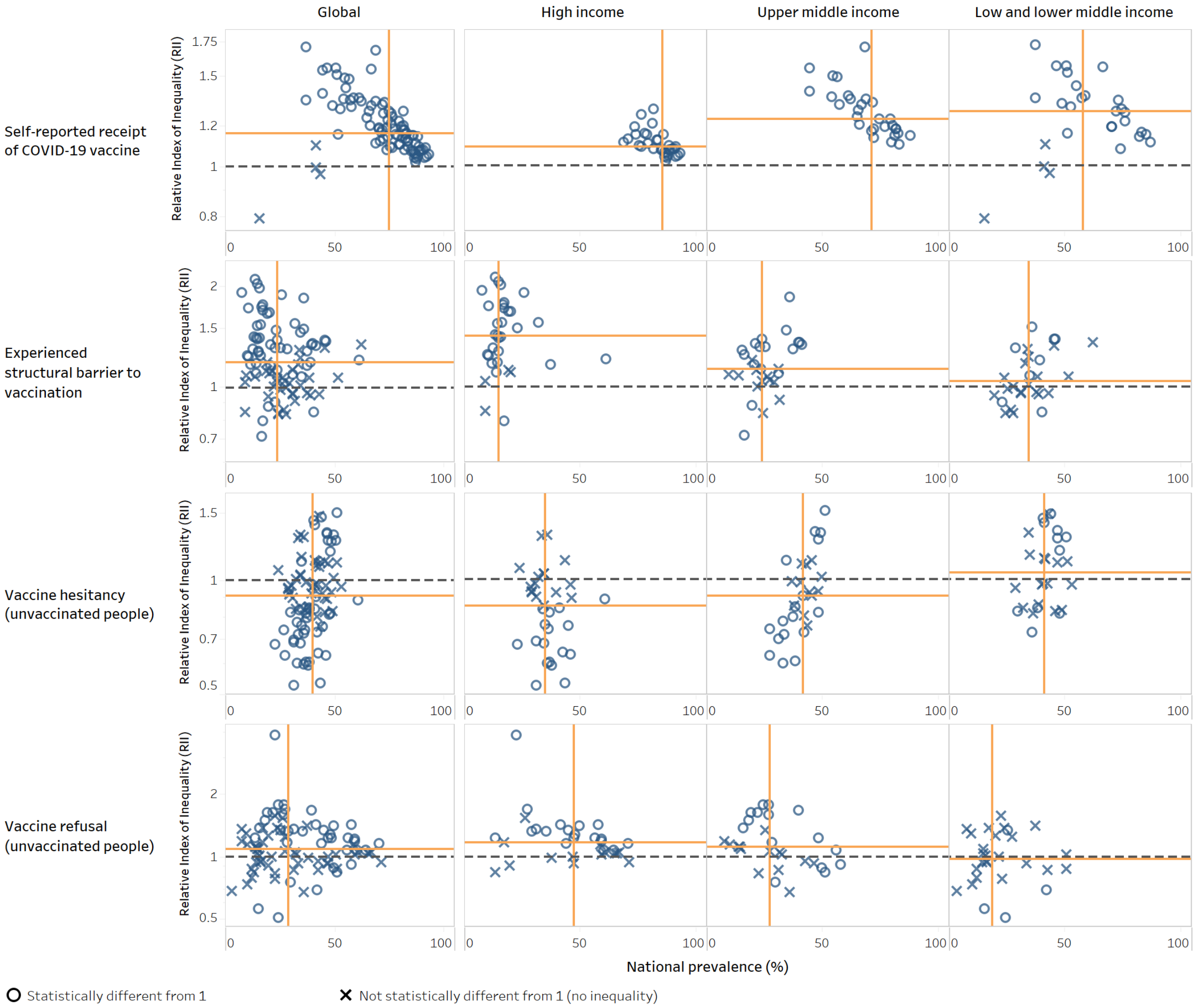

RII: relative index of inequality. UMD Global CTIS: University of Maryland Social Data Science Center Global COVID-19 Trends and Impact Survey.
Notes: Blue symbols represent countries. Orange lines show the medians across countries. Black dashed line shows no inequality (zero). RII of 1 indicates no inequality; RII above 1 denotes higher indicator prevalence among people with more education, and RII below 1 denotes higher indicator prevalence among people with less education. RII is adjusted for individual sociodemographic characteristics (age, gender, place of residence, and household overcrowding), the presence of health risk factors and COVID-like symptoms.

Figure S3. Education-related inequality in experience of structural barriers to vaccination: unadjusted and adjusted slope index of inequality in 90 study countries (UMD Global CTIS, June–December 2021)

Unadjusted Slope Index of Inequality

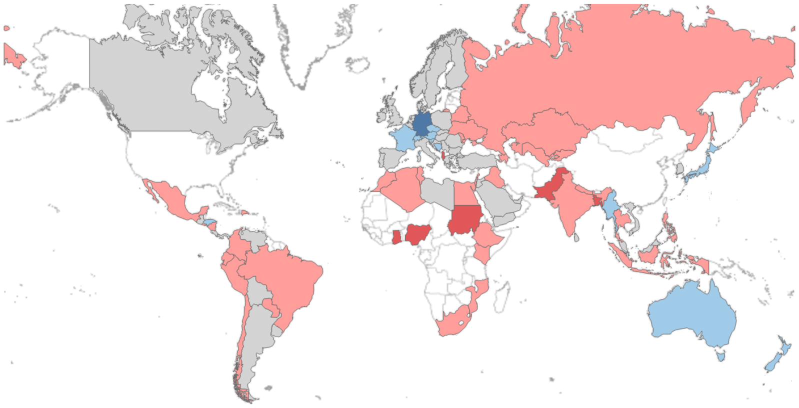

© 2022 Mapbox © OpenStreetMap

Adjusted Slope Index of Inequality

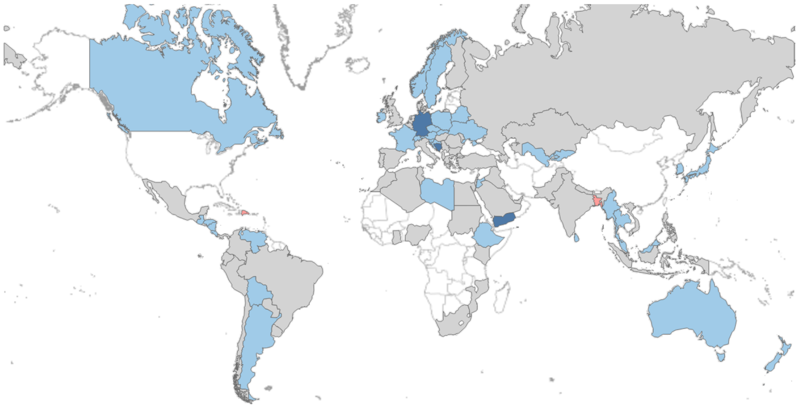

© 2022 Mapbox © OpenStreetMap

- High inequality (higher prevalence among the most educated)
- Moderate inequality (higher prevalence among the most educated)
- Low inequality
- Moderate inequality (higher prevalence among the least educated)
- High inequality (higher prevalence among the least educated)

SII: Slope index of inequality. UMD Global CTIS: University of Maryland Social Data Science Center Global COVID-19 Trends and Impact Survey.

Notes: High inequality denotes an absolute SII of 15 percentage points or more; moderate inequality denotes an absolute SII of 5-15 percentage points; low inequality denotes an absolute SII of less than 5 percentage points. SII is adjusted for individual sociodemographic characteristics (age, gender, place of residence, and household overcrowding), the presence of health risk factors and COVID-like symptoms.

Table S1. Education-related inequality in COVID-19 vaccine indicators: adjusted slope index of inequality (percentage points) and relative index of inequality, globally and by country income group for males and females (UMD Global CTIS, June–December 2021)

			Adjusted slope index of inequality (95% CI)		Adjusted relative index of inequality (95% CI)	
**Indicator**	**Income group**	**No· countries**	**Male**	**Female**	**Male**	**Female**
Self-reported receipt of COVID-19 vaccine	Global	90	-11·9 (-13·4–-9·9)	-11·6 (-12·9–-10·1)	0·85 (0·82–0·88)	0·85 (0·82–0·88)
	High income	33	-7·4 (-9·2–-5·7)	-7·0 (-8·6–-4·7)	0·91 (0·89–0·94)	0·92 (0·89–0·95)
	Upper middle income	29	-15·3 (-18·2–-13·1)	-14·8 (-16·6–-12·1)	0·82 (0·75–0·83)	0·82 (0·77–0·85)
	Low and lower middle income	28	-13·9 (-18·5–-9·5)	-13·3 (-18·3–-11·5)	0·79 (0·75–0·86)	0·78 (0·73–0·85)
Experienced structural barrier to vaccination	Global	90	-3·6 (-5·4–-2·4)	-4·4 (-5·2–-2·6)	0·85 (0·80–0·91)	0·81 (0·73–0·87)
	High income	33	-5·0 (-7·5–-3·3)	-5·2 (-9·0–-4·5)	0·76 (0·65–0·82)	0·66 (0·60–0·76)
	Upper middle income	29	-3·2 (-6·6–-1·4)	-3·8 (-7·4–-2·1)	0·90 (0·78–0·95)	0·83 (0·72–0·91)
	Low and lower middle income	28	-1·3 (-5·5–0·3)	-1·6 (-4·2–1·4)	0·95 (0·88–1·01)	0·95 (0·89–1·06)
Vaccine hesitancy (unvaccinated people)	Global	90	-2·0 (-3·7–-0·2)	-0·5 (-1·9–1·1)	0·98 (0·96–1·00)	0·99 (0·97–1·02)
	High income	33	-2·1 (-3·7–0·0)	-1·2 (-2·3–0·6)	0·98 (0·96–1·00)	0·99 (0·97–1·01)
	Upper middle income	29	-1·9 (-6·3–1·7)	1·9 (-2·8–5·3)	0·96 (0·93–1·03)	1·02 (0·97–1·06)
	Low and lower middle income	28	-2·2 (-8·0–2·2)	-1·7 (-10·9–4·4)	0·97 (0·89–1·03)	0·97 (0·86–1·09)
Vaccine refusal (unvaccinated people)	Global	90	-5·8 (-7·4–-4·1)	-1·9 (-4·6–0·6)	0·83 (0·78–0·90)	0·96 (0·89–1·01)
	High income	33	-9·8 (-13·3–-6·3)	-5·1 (-8·9–-2·1)	0·83 (0·73–0·88)	0·90 (0·78–0·96)
	Upper middle income	29	-6·3 (-10·6–-2·6)	0·6 (-4·3–2·2)	0·74 (0·68–0·92)	1·02 (0·77–1·13)
	Low and lower middle income	28	-1·6 (-4·0–0·8)	1·0 (-3·0–3·6)	0·91 (0·83–1·08)	1·04 (0·82–1·24)

CI: Confidence interval. UMD Global CTIS: University of Maryland Social Data Science Center Global COVID-19 Trends and Impact Survey. Notes: Median values in low and lower middle income countries for females based on 26 countries (experienced structural barrier), 27 countries (vaccine hesitancy) and 25 countries (vaccine refusal). SII and RII express the difference and ratio, respectively, between two extremes of the education distribution, accounting for the situation across the population. SII of 0 (RII of 1) indicates no inequality; SII above 0 (RII above 1) denotes higher indicator prevalence among people with more education, and SII below 0 (RII below 1) denotes higher indicator prevalence among people with less education. SII and RII are adjusted for individual sociodemographic characteristics (age, place of residence, and household overcrowding), the presence of health risk factors and COVID-like symptoms.

Table S2. COVID-19 vaccine indicators by education level: median relative index of inequality (RII), globally and by country income group (UMD-CTIS, June–December 2021)

			Median relative index of inequality (95% CI)		
**Indicator**	**Income group**	**No· countries**	**Unadjusted**	**Adjusted**	**Adjusted2**
Self-reported receipt of COVID-19 vaccine	Global	90	1·26 (1·19–1·30)	1·15 (1·13–1·20)	1·16 (1·14–1·20)
	High income	33	1·12 (1·09–1·17)	1·09 (1·06–1·12)	1·09 (1·07–1·12)
	Upper middle income	29	1·36 (1·27–1·49)	1·21 (1·19–1·32)	1·23 (1·18–1·33)
	Low and lower middle income	28	1·39 (1·28–1·55)	1·25 (1·16–1·33)	1·27 (1·16–1·35)
Experienced structural barrier to vaccination	Global	90	0·86 (0·81–0·94)	1·19 (1·13–1·28)	1·20 (1·12–1·29)
	High income	33	1·13 (1·02–1·30)	1·36 (1·21–1·62)	1·41 (1·24–1·63)
	Upper middle income	29	0·78 (0·70–0·86)	1·18 (1·10–1·34)	1·13 (1·08–1·30)
	Low and lower middle income	28	0·76 (0·68–0·84)	1·02 (0·95–1·14)	1·04 (0·96–1·19)
Vaccine hesitancy (unvaccinated people)	Global	90	0·90 (0·85–0·98)	0·89 (0·84–0·92)	0·91 (0·84–0·97)
	High income	33	0·84 (0·76–0·92)	0·83 (0·75–0·89)	0·85 (0·74–0·93)
	Upper middle income	29	0·86 (0·77–1·02)	0·89 (0·78–0·96)	0·90 (0·79–0·99)
	Low and lower middle income	28	1·03 (0·94–1·19)	1·05 (0·91–1·14)	1·04 (0·88–1·18)
Vaccine refusal (unvaccinated people)	Global	90	1·22 (1·14–1·30)	1·13 (1·09–1·19)	1·09 (1·04–1·18)
	High income	33	1·24 (1·14–1·34)	1·17 (1·11–1·29)	1·18 (1·06–1·26)
	Upper middle income	29	1·29 (1·09–1·61)	1·13 (1·00–1·45)	1·12 (1·00–1·27)
	Low and lower middle income	28	1·16 (0·97–1·34)	1·07 (0·94–1·17)	0·98 (0·87–1·20)

CI: confidence interval. RII: relative index of inequality. UMD-CTIS: University of Maryland Social Data Science Center Global COVID-19 Trends and Impact Survey.
Notes: Medians are based on countries with sample sizes of at least 100 in each education subgroup. RII expresses the ratio between two extremes of the education distribution, accounting for the situation across the population. RII of 1 indicates no inequality; RII above 1 denotes higher indicator prevalence among people with more education, and RII below 1 denotes higher indicator prevalence among people with less education. The first adjusted model (Adjusted) controlled for individual sociodemographic characteristics (age, gender, place of residence, and household overcrowding). The second adjusted model (Adjusted2) controlled for these characteristics plus the presence of health risk factors and COVID-like symptoms.

Table S3. Associations between government vaccine availability policies and COVID-19 vaccine indicators (national prevalence and education-related inequality) (UMD-CTIS, June–December 2021)

		National prevalence		Education-related inequality	
**Indicator**	**Income group**	**Correlation coefficient**	**P-value**	**Correlation coefficient**	**P-value**
Self-reported receipt of COVID-19 vaccine	Global	0·568	<0·000	-0·477	<0·000
	High income	0·471	<0·000	-0·303	<0·000
	Upper middle income	0·370	<0·000	-0·424	<0·000
	Low and lower middle income	0·549	<0·000	-0·404	<0·000
Vaccine hesitancy (unvaccinated people)	Global	-0.208	<0.000	*	*
	High income	-0.092	0.165	0.068	0.303
	Upper middle income	-0.092	0.191	0.035	0.625
	Low and lower middle income	-0.042	0.542	*	*
Vaccine refusal (unvaccinated people)	Global	0.567	<0.000	*	*
	High income	0.391	<0.000	0.027	0.680
	Upper middle income	0.609	<0.000	0.058	0.408
	Low and lower middle income	0.272	<0.000	*	*

UMD-CTIS: University of Maryland Social Data Science Center Global COVID-19 Trends and Impact Survey.
Notes: Education-related inequality measured using crude (unadjusted) slope index of inequality. An ordinal scale was applied to reflect the country-level government policies for vaccine availability, specifying three population groups (key workers, clinically vulnerable people or the elderly): 0 indicates no availability to any group; 1 indicates availability for one of the key groups; 2 indicates availability for two of the key groups; 3 indicates availability for all three key groups; 4 indicates availability for all three key groups plus partial additional availability; and 5 indicates universal availability for the entire population.
*Results not reported when country-level slope index of inequality (SII) values included both negative and positive values.
